# Assessment of Healthcare Professionals' Knowledge Regarding Nontuberculosis Mycobacterial Infections at Debre Tabor Comprehensive Specialized Hospital, Northwest Ethiopia, 2024: A Cross‐Sectional Study

**DOI:** 10.1002/jcla.70035

**Published:** 2025-04-18

**Authors:** Teklehaimanot Kiros, Mulat Erkihun, Bekele Sharew, Andargachew Almaw, Ayenew Assefa, Tegenaw Tiruneh, Birhanu Getie, Yenealem Solomon, Shewaneh Damtie

**Affiliations:** ^1^ Department of Medical Laboratory Sciences, College of Health Sciences Debre Tabor University Debre Tabor Ethiopia

**Keywords:** Ethiopia, healthcare professionals, knowledge, nontuberculous mycobacteria

## Abstract

**Background:**

Nontuberculosis mycobacteria (NTM) are environmental pathogens that can cause pulmonary infections, especially among the immunocompromised population. There is limited research on healthcare professionals' knowledge of NTM infections in Ethiopia. This study aimed to evaluate healthcare professionals' knowledge about NTM infections at Debre Tabor Comprehensive Specialized Hospital (DTCSH).

**Methods:**

A cross‐sectional study was conducted from March 5 to July 30, 2024 at DTCSH to evaluate the knowledge of 292 healthcare professionals on NTM infections. A semi‐structured questionnaire was used for data collection, and the data were analyzed using SPSS version 25. The study's findings were presented through texts, tables, and figures.

**Results:**

Of all the participants, 160 (54.8%) were male, and the majority 153 (52.4%) were 36–45 years old. Nurses made up the largest professional group, 127 (43.5%). Approximately 59% of healthcare professionals showed good knowledge of NTM infections. In this study, a higher percentage of female [92 (69.7%)] participants demonstrated good knowledge compared to males [83 out of 160 males (51.9%)]. Similarly, 35 doctors (89.7%) and 80 nurses (63%) demonstrated favorable knowledge. Only 52.4% could distinguish between NTM and *Mycobacterium tuberculosis* infections. Notably, 77.4% recognized that NTM infections are more common in immunocompromised individuals, yet only 20.5% knew that NTM species are not typically treated with anti‐tuberculosis drugs.

**Conclusion:**

The knowledge gaps among healthcare professionals regarding NTM infections necessitate focused educational initiatives to improve their ability to manage these infections. The focused training is essential for enhancing the management and identification of NTM infections.

## Graphical abstract

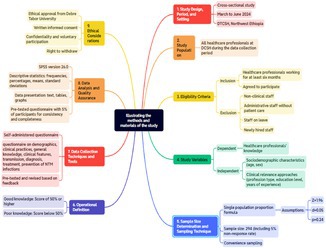

## 1 Introduction

Nontuberculosis mycobacteria (NTM) are environmental opportunistic human pathogens commonly found in peat‐rich potting soil and water in buildings and households. They belong to mycobacterial species that are not part of the *Mycobacterium tuberculosis* (MTB) complex or *Mycobacterium leprae* . Due to their ubiquitous presence in the environment, humans can acquire NTM infections through inhalation of contaminated sources [1, 2, 3]. Mycobacterial infections, including those caused by NTM other than the *Mycobacterium avium* complex (MAC), have assumed greater importance over the past decade due to the changing spectrum of immunosuppression associated with organ transplantation. Various pathogenic NTMs have been identified and are associated with a wide variety of localized organ‐specific and systemic infections [4, 5]. Human infection is mostly caused by the slow‐growing MAC, which now includes MAC subspecies silvaticum, hominissuis, and paratuberculosis, among others. Other common NTM isolated from human samples include *Mycobacterium xenopi* , *Mycobacterium fortuitum* complex, *Mycobacterium kansasii* , and the rapidly growing *Mycobacterium abscessus* group (MABS), recently grouped as a separate clade named *Mycobacteriodes abscessus* based on phylogenetic characteristics [3, 6, 7].

The global incidence of NTM infections is increasing, especially in developing countries, where socio‐economic factors, clinical data, and disease origins play a significant role. These infections are more prevalent among individuals with preexisting pulmonary diseases and those with compromised immune systems, although they can also occur in people without any apparent risk factors [8]. The epidemiology of NTM revealed variations in prevalence and incidence in various geographic regions, where NTM infections are increasingly recognized as significant contributors to pulmonary disease, often complicating the diagnosis and treatment of TB [9]. NTM infections are rising globally, with significant increases reported in Asia, particularly in Japan, South Korea, and Taiwan [10]. In the United States, a multicenter study indicated a decrease in hospital‐onset NTM infections from 2014 to 2020, yet variability among hospitals persisted [11]. A study in Nigeria has revealed NTM prevalence of 8.8% [12]. Similarly, another study on the analysis of NTM in high endemic areas of TB found that the prevalence of co‐infection with NTM and TB was 4.2% in TB patients and 7.6% in NTM patients, highlighting the significant epidemiological overlap [13]. The prevalence of NTM infections in Ethiopia reveals an important public health issue, particularly among vulnerable populations such as those with HIV. The prevalence of NTM infections is notably high, often misdiagnosed as tuberculosis, complicating treatment and management strategies. Based on the systematic review and meta‐analysis conducted in sub‐Saharan Africa, the prevalence of pulmonary NTM was 7.5% [14]. Co‐infection of NTM with MTB complicates clinical presentations and leads to misdiagnosis. While the rising prevalence of NTM poses challenges for public health, it also highlights the need for enhanced diagnostic capabilities and treatment strategies to differentiate between NTM and TB effectively. This dual burden necessitates a comprehensive approach to managing both infections in clinical settings [8]. Most studies report an increase in pulmonary NTM infections (82.1%) and diseases (66.7%) globally. The annual rate of change for NTM infection and disease is 4.0% and 4.1%, respectively [15]. The prevalence of NTM infections is notably increasing in cystic fibrosis populations, with a pooled estimate of 7.9% [16].

The transmission of NTM is similar to that of tuberculosis (TB) through aerosol. However, NTM transmission sources are environmental rather than human or animal, suggesting that pulmonary NTM (PNTM) is not contagious. The likelihood of acquiring PNTM increases with exposure to environmental factors associated with water and soil. The rise of PNTM can be attributed to advancements in mycobacteria detection methods and multifactorial factors stemming from the pathogen, host, and host‐pathogen interactions, as well as inadequate disease management [17, 18, 19]. NTM can lead to localized or disseminated diseases depending on local predispositions and/or immune deficits. Among people living with HIV, various NTM strains can cause localized pulmonary disease, adenitis, soft tissue infections, joint and bone infections, bursitis, skin ulcers, and generalized disease in individuals such as leukemia or transplant patients [5, 20, 21].

The identification of these organisms in pulmonary specimens does not always indicate active infection; supportive radiographic and clinical findings are needed to establish the diagnosis [22, 23, 24]. NTM are susceptible to harsh decontamination techniques, making detection difficult for fastidious species like *Mycobacterium haemophilum* and those requiring incubation at 30°C (e.g., *Mycobacterium marinum* ) or 45°C ( *Mycobacterium xenopi* ), as well as extremely slow‐growing species like *M. ulcerans* [7, 25]. The drug susceptibility profile of NTM is usually quite different from MTB. These organisms are usually sensitive at high concentrations of antituberculosis drugs; thus, higher cut‐off values for deciding sensitivity/resistance are recommended. Rapid‐growing mycobacteria are usually resistant to rifampicin and isoniazid (INH) but sensitive to new‐generation macrolides and cephalosporins [7, 26, 27].

Controlling NTM infection can be achieved in part by significantly raising awareness among healthcare workers to heighten their suspicion of patients presenting at their hospitals. Maintaining a high index of suspicion for presumptive TB cases and cases under treatment is crucial for ruling out NTM and effectively managing the cases. Healthcare professionals encountering NTM infections should be knowledgeable about the different species of NTM, their clinical presentation, diagnostic methods (such as culturing and molecular testing), and treatment options. Due to the difficulties in diagnosing and treating NTM infections, healthcare professionals often collaborate with microbiologists and infectious disease specialists to ensure accurate diagnosis and proper treatment [1, 28, 29].

Limited research has been conducted on the knowledge of healthcare professionals regarding non‐NTM infections in Ethiopia. This study is significant for enhancing the quality of care, improving patient outcomes, and advancing public health efforts to address this neglected yet important cause of pulmonary disease in the country. Many healthcare professionals, including physicians, nurses, and laboratory staff, may have a limited understanding of the epidemiology, clinical presentation, diagnostic methods, and treatment options for NTM infections [1, 23]. This knowledge gap can lead to under‐recognition of NTM infections, inappropriate use of antibiotics, treatment failures, and increased healthcare costs. This study emphasized the need for increased awareness, improved diagnostic capacity, and updated treatment guidelines for NTM infections among healthcare professionals. Furthermore, the rising prevalence of NTM infections, particularly in immunocompromised individuals and those with underlying lung conditions such as bronchiolitis and cystic fibrosis, underscores [21, 30] the importance of improving healthcare professionals' knowledge and expertise in managing these challenging infections [6, 21]. Currently, there are no published studies on this health topic. This cross‐sectional survey aims to provide the first insights into the knowledge state among healthcare professionals, specifically in our setting and generally in Ethiopia. Therefore, this study aimed to assess the knowledge of NTM infection among healthcare professionals at Debre Tabor Comprehensive Specialized Hospital (DTCSH). Given the complexity and multifaceted nature of NTM infections, this assessment is crucial for developing effective educational initiatives, diagnostic strategies, and treatment protocols to enhance the overall quality of care provided to patients with NTM infections.

## 2 Methods and Materials

### 2.1 Study Design, Period, and Setting

A cross‐sectional study was conducted from March 5 to July 30, 2024, to assess the knowledge of healthcare professionals regarding NTM infection at DTCSH in Northwest Ethiopia. DTCSH is a key tertiary health facility located in the capital of the South Gondar Zone, Amhara region, Ethiopia. The hospital is recognized for improving health outcomes through clinical excellence, community involvement, and research. It addresses key health challenges, including maternal and child health, infectious diseases, and chronic conditions. In collaboration with Debre Tabor University, the hospital ensures high‐quality care from skilled professionals, supported by ongoing research and professional development. The South Gondar zone encompasses 405 health posts, 96 health facilities, eight basic hospitals, and one comprehensive specialized hospital. It includes four town administrations and 14 districts. DTCSH employs over 600 healthcare professionals and has more than 300 beds in various wards, including pediatric, medical, surgical, and psychiatric units. Debre Tabor is located at a latitude of 11.850° N, a longitude of 38.017° E, and an elevation of 2706 m (8,878 ft) above sea level.

### 2.2 Study Population

All healthcare professionals working at DCSH during the data collection period were considered as the study population.

### 2.3 Eligibility Criteria

The inclusion criteria cover all healthcare professionals who have been working at the hospital for at least 6 months and who agreed to take part in the study. However, non‐clinical staff who were not directly involved in patient care, as well as individuals without formal education or training in healthcare, were not included. Healthcare professionals who were on leave during the data collection period as well as newly hired staff were also excluded.

### 2.4 Sample Size Determination and Sampling Technique

The sample size for this study was calculated using a single population proportion formula using the following assumptions:

$$n=\frac{Z_{a/2}^{2}\times p\times \left(1-p\right)}{d^{2}}$$

where *n* = total sample size, *z* = the standard normal variation at 95% confidence interval, *α* = 1.96, *d* = margin of error (*d* = 0.05), *p* = the proportion of the population with knowledge from the previous study (24.1%) [1], then, *n* = (1.96)^2^ × 0.24(0.76)/(0.05)^2^, *n* = 280.

Adding 5% of the non‐response rate, 280 × 5/100 = 14, the total sample size = 280 + 14 = 294.

The 5% non‐response rate is a reasonable assumption that accounts for minor participant dropout without excessively inflating the sample size. This approach is based on common practices in research and effective resource management. It represents a realistic and attainable expectation for study participation while maintaining the study's overall efficiency.

Convenience sampling was used to choose participants for this study. Based on the aim of the study, a variety of healthcare professionals, including clinicians, nurses, laboratory technologists, pharmacists, midwives, and other healthcare professionals working in different departments were conveniently selected.

### 2.5 Operational Definition

For this study, a 29‐item questionnaire was used to assess the knowledge of healthcare professionals regarding NTM infections. Each participant's score was out of a total of 29, which was then multiplied by 100 to obtain a percentage score. Then, the knowledge level of participants was operationalized as follows:

1. *Good knowledge*: Participants who scored 50% or higher on all knowledge questions. In closed‐ended questionnaires, each correct response was assigned a score of 1, while an incorrect or uncertain response was scored as 0. In open‐ended or multiple‐choice questionnaires, participants who provided at least one appropriate answer were considered knowledgeable [1].
2. *Poor knowledge*: A score of 0 to 49% was considered poor/unfavorable knowledge [1].
3. *A healthcare professional* is defined as an individual engaged in activities aimed at enhancing health, such as physicians, nurses, and other professionals involved in patient care within the health care sector.

### 2.6 Data Collection Techniques and Tools

**FIGURE 1:**
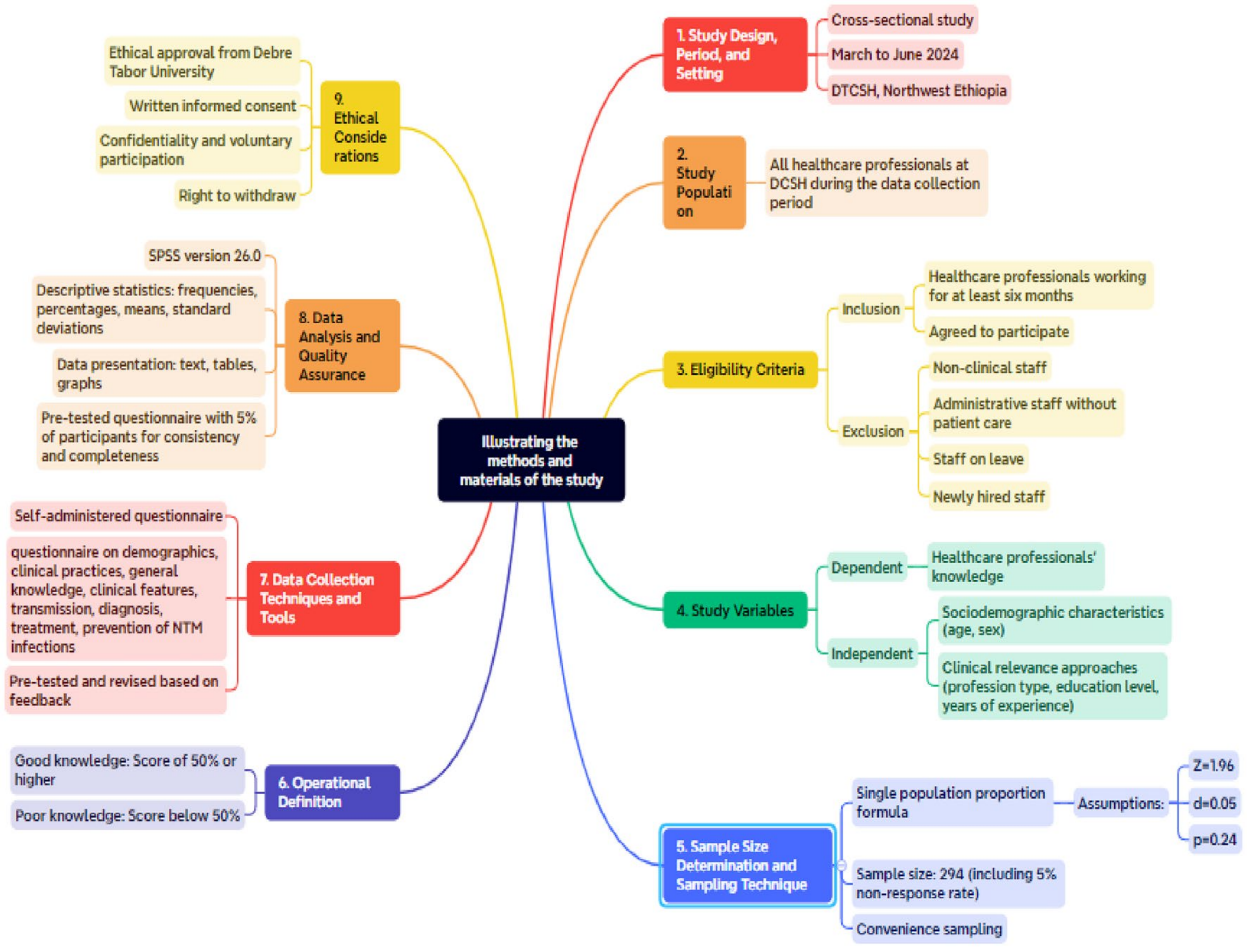
A comprehensive and schematic illustration of the study's methods and materials.

Prior to collecting data, participants were given written consent and an information sheet to ensure their voluntary participation. A well‐structured, self‐administered questionnaire was developed by designated authors to gather important data based on a comprehensive review of the literature, including the World Health Organization (WHO) guideline towards knowledge, attitude, and practice [31], and included questions on demographic information, clinical practices, general knowledge, clinical features, transmission, diagnosis, treatment, and prevention of NTM infections. The questionnaire was pre‐tested on a small sample of healthcare professionals working outside of the study facility (other district hospitals in South Gondar) selected from various departments/wards to ensure that it was clear, complete, and simple. Based on the feedback received, necessary modifications were made. Trained data collectors (nurses) distributed the questionnaires to the selected healthcare professionals and ensured that they had enough time to complete them. The participants were guaranteed the confidentiality of their responses. The overall activity of this study is illustrated in Figure 1.

### 2.7 Data Analysis and Data Quality Assurance

The data were entered into SPSS version 26.0 and cleaned for statistical analysis. Descriptive statistics, such as frequencies, and percentages were used to summarize the data, which was presented using text, tables, and figures. To ensure data quality, the questionnaire was carefully reviewed and modified based on the authors' knowledge, experience, and expertise in the field. Prior to actual data collection, the questionnaire was pre‐tested among healthcare workers in surrounding district hospitals to ensure consistency and completeness. After the pre‐test, the questionnaire was revised to rephrase questions with multiple interpretations, ensuring they were specific and used simple language to minimize biases as per standard requirements [31].

### 2.8 Ethical Considerations

Ethical approval for the study was obtained from Debre Tabor University's ethical review committee (Reference number: CHS1849/2024). After explaining the purpose of the study, ensuring confidentiality, and assuring that participation was voluntary, written informed consent was obtained from each participant. The process was conducted using the participant's study identification number to maintain anonymity. Participants had the right to withdraw from the study at any time without consequences.

## 3 Results

Two participants were withdrawn from this study due to personal matters. The overall response rate for this study was 99.3% (292/294). Most participants were male 160 (54.8%), aged between 36 and 45 years 153 (52.4%), and held a Bachelor's degree 150 (51.4%). The mean age of the healthcare professionals was 37 years (37 ± 1.87) with a range between 23 and 55 years. Nurses formed the largest professional group 127 (43.5%), and the majority had 11–15 years of work experience 116 (39.7%) as shown in (Table 1).

**TABLE 1:** The sociodemographic characteristics of the healthcare professionals (*n* = 292) working at Debre Tabor Comprehensive Specialized Hospital, Northwest Ethiopia 2024.

R. No	Study variables	Category	Frequency (*N*)	Percentage (%)
1	Gender	Male	160	54.8
		Female	132	45.2
2	Age (years)	18–25	2	0.7
		26–35	101	34.6
		36–45	153	52.4
		46–55	36	12.3
3	Level of education	Diploma	66	22.6
		Bachelor degree	150	51.4
		Master's degree	52	17.8
		Doctorate	24	8.2
	Health profession	Doctor	39	13.4
		Medical lab	33	11.3
		Nurses	127	43.5
		Pharmacy	19	6.5
		Midwife	46	15.8
		Other^a^	28	9.6
5	Work experience (years)	1–5	39	13.4
		6–10	70	24.0
		11–15	116	39.7
		≥ 16	67	22.9

^a^ Psychiatry, anesthesia, radiology, dental, optometry.

### 3.1 Knowledge Distribution of Healthcare Professionals

**FIGURE 2:**
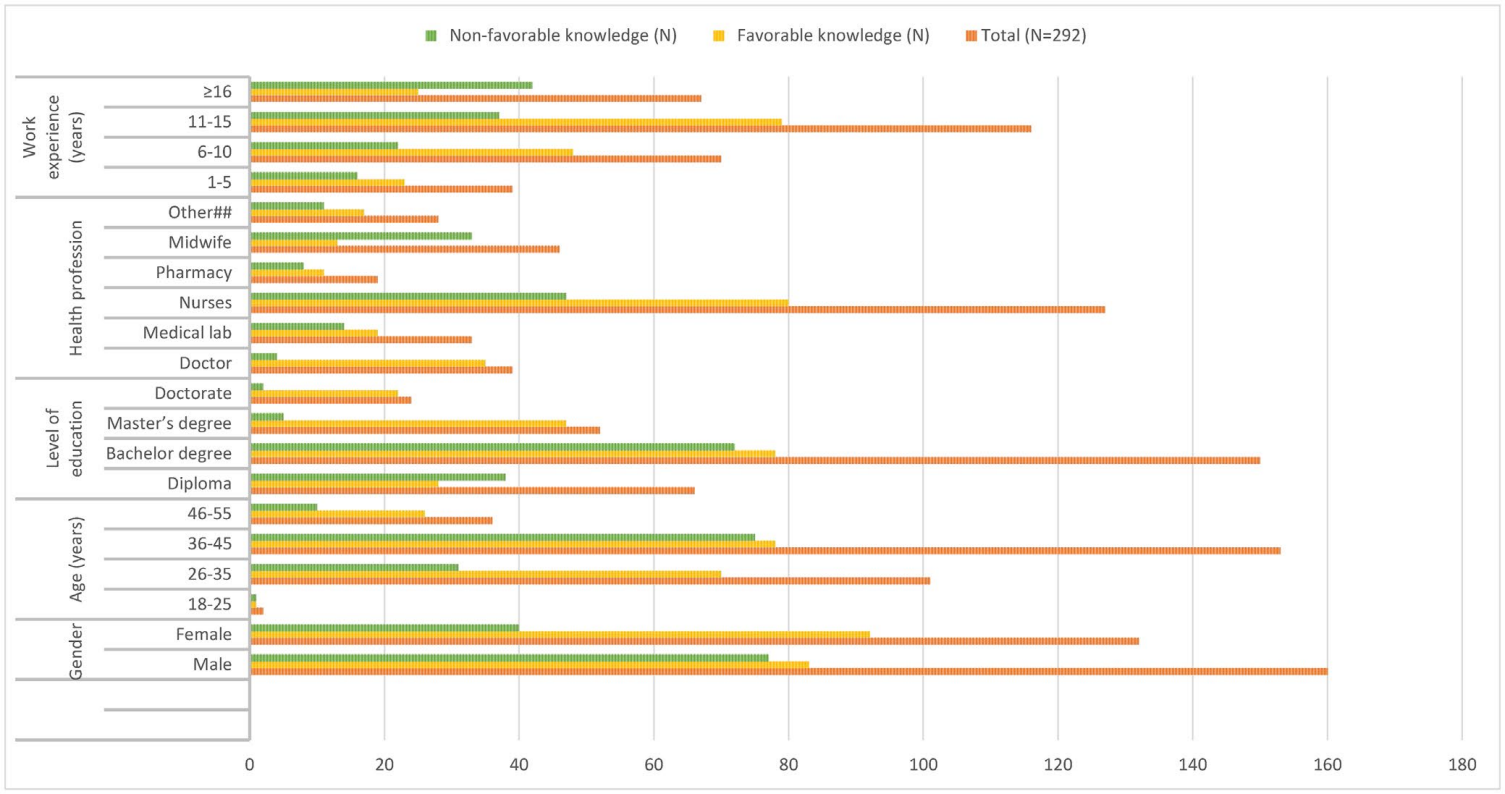
Knowledge status distribution of healthcare professionals based on the study variables.

The knowledge characteristics of healthcare professionals varied significantly based on gender, age, education level, profession, and work experience (Figure 2). In this study, a higher percentage of female [92 (69.7%)] participants demonstrated good knowledge compared to males [83 out of 160 males (51.9%)]. Healthcare professionals aged 26–35 and 36–45 had the highest levels of favorable knowledge, with 69.3% and 51%, respectively. Looking at the different health professions, about 35 out of 39 doctors (89.7%) and 80 out of 127 nurses (63%) demonstrated favorable knowledge, whereas only 13 out of 46 midwives (28.3%) were knowledgeable. In terms of work experience, professionals with 11–15 years of experience were the most knowledgeable [79 out of 116 (68.1%) showed favorable knowledge]. In contrast, those with over 16 years of experience had only 37.3% favorable knowledge.

### 3.2 Knowledge Status of Healthcare Professionals Regarding NTM Infections

The knowledge level was assessed using a 29‐item questionnaire predominantly structured into evaluating general knowledge, transmission dynamics, laboratory diagnosis, treatment, and control strategies of pulmonary NTM infections. Out of the total, around 175 of the healthcare professionals answered at least 17 out of 29 questions correctly. The results showed that approximately 175 (59%) participants demonstrated favorable knowledge of NTM infections. Similarly, the findings showed that around 52.4% of participants could distinguish between NTM and MTB infections (Table 2). Additionally, 46.2% recognized the clinical differences between pulmonary NTM and TB. The majority of respondents (97.6%) have not encountered NTM patients, and a significant portion (99.0%) have not met patients at risk for NTM. Approximately 44% knew the common symptoms, but only 18.8% have discussed pulmonary NTM infections with their colleagues. Only 36.6% of the participants understood that airborne droplets are a primary transmission route, while 81.8% considered that GeneXpert could detect both NTM and MTB.

**TABLE 2:** Assessing the knowledge status of healthcare workers towards NTM infections.

Category	Specific questions	Response/opinions	Frequency (*N*)	Percentage (%)
General	1. Do you know about NTM	Yes	236	80.8
		No	56	19.2
	2. Do you know the difference between NTM and MTB infection	Yes	153	52.4
		No	139	47.6
	3. Are pulmonary NTM and TB clinically different?	Yes	135	46.2
		No	157	53.8
	4. Source of NTM infection in healthcare settings	Contaminated water	51	17.5
		Person‐to‐person contact	115	39.4
		Hospital food	49	16.8
		Not sure	75	25.7
	5. Have you encountered a patient with NTM infections in your practice?	Yes	7	2.4
		No	285	97.6
	6. How many cases have you encountered	Nope	287	98.3
		6–10 case	2	0.7
		More than 10 case	3	1.0
	7. Have you encountered patients with risk factors for NTM infections in your practice	Yes	3	1.00
		No	289	99.0
	8. What are the common symptoms of NTM infections	Fever	5	1.7
		Weight loss	15	5.1
		Shortness of breath	7	2.4
		All of the above	128	43.8
	9. Have you ever discussed about NTM with your colleague	Yes	55	18.8
		No	237	81.2
	10. How important is NTM infections for healthcare professionals	Very important	245	83.9
		Somewhat important	44	15.1
		Not sure	1	0.3
Transmission	1. Are NTM freely available in water and soil	Yes	214	73.3
		No	78	26.7
	2. Do you know the transmission vehicles of NTM	Yes	174	59.6
		No	118	40.4
	3. Possible sources of NTM infections	Air born droplet	107	36.6
		Contaminated food and water	14	4.8
		Direct contact with infected individuals	55	18.8
		Animal contact	116	39.7

	4. Can NTM be transmitted from one person to another	Yes	135	46.2
		No	157	53.8
	5. Participate in research projects related to NTM infection control	Yes	13	4.5
No		279	95.5
	6. NTM infections can be transmitted by contaminated medical devices and inanimate surfaces	Yes	186	63.7
		No	52	17.8
		Not sure	54	18.5
Diagnosis	1. How is NTM infection diagnosed?	Microscopy	132	45.2
		Chest x‐ray/CT scan	47	16.1
		Sputum culture	98	33.6
		Others (GeneXpert)	15	5.1
	2. Can microscopy distinguish between MTB and NTM	Yes	118	40.4
		No	174	59.6
	3. Can GeneXpert detect both NTM and MTB	Yes	239	81.8
		No	53	18.2
	4. Challenges associated with diagnosing NTM infections in the laboratory	Contaminated samples	13	4.5
		Slow growth of NTM on culture	51	17.5
		Lack of specific diagnostic test	36	12.3
		Difficulty in distinguishing NTM from TB	70	24.0
		All	122	41.8
Prevention and Treatment	1. Are NTM more common in immunocompromised individuals	Yes	226	77.4
		No	66	22.6
	2. Can NTM species be treated with TB drugs	Yes	60	20.5
		No	232	79.5
	3. Have you encountered patients with treatment failure or relapse for NTM infections	Yes		3.1
		No	283	96.9
	4. What is the first‐line treatment for most NTM infections	Isoniazid	23	7.9
		Rifampin	93	31.8
		Ethambutol	13	4.5
		Clarithromycin	41	14.0
		Not sure	120	41.1
	5. Recommended treatment duration for NTM infections	1 Week	18	6.2
		1 Month	42	14.4
		6 Month to 2 Years	123	42.1
		Indefinite	109	37.3

	6. NTM infections require multiple antibiotics and can be prolonged.	Yes	215	73.6
		No	77	26.4
	7. Is Previous TB exposure risk for NTM?	Yes	88	30.1
		No	204	69.9
	8. Measures for the prevention of NTM infections in healthcare settings	Environmental sanitation	29	9.9
		Food hygiene	70	24.0
		Use of PPE	18	6.2
		Hand hygiene practice	164	56.1
		Patient isolation	11	3.8
	9. Have you received training on infection control measures specific to NTM infections	Yes	10	3.4
No		282	96.6

## 4 Discussion

NTMs are environmental agents that can cause opportunistic pulmonary disease in humans and animals. These infections commonly occur in patients with pre‐existing lung diseases or reduced immunity. A knowledge gap, poor awareness, and practice regarding NTM infections among healthcare professionals may affect the quality of healthcare [6, 7]. To make a significant impact, it is important to improve knowledge within the community, particularly among healthcare providers. This can be achieved through targeted educational initiatives, related training in continuous professional development (CPD), and raising awareness about disease transmission, diagnosis, treatment, and infection prevention and control [17, 23, 29, 32].

In this study, 59% of healthcare professionals have shown favorable knowledge regarding NTM infections. Only 24.1% of healthcare workers (HCWs) in Tanzania were found to be knowledgeable about NTM infections, indicating a substantial knowledge gap that may have an impact on diagnosis and treatment [1]. A study conducted across a network of 10 hospitals in the United States of America showed that 24% of NTM infection episodes began in the hospital [11]. A French study linked medical and cosmetic interventions to increased NTM infections, emphasizing the need for HCWs to follow strict infection control protocols [33]. According to a Saudi Arabian survey, 64% of HCWs had adequate knowledge, but they also pointed out areas where their practical application was lacking, which emphasizes the need for better training initiatives [34]. Although 59% of HCWs in Pakistan knew about TB, there were gaps in their knowledge due to inadequate infrastructure and training [35]. These disparities can be ascribed to many factors, such as variations in healthcare systems, resource accessibility, educational initiatives, and socioeconomic circumstances. These gaps should be remedied with specialist training, improved facilities, and specially created educational programs to reduce infection transmissions in the community and in the healthcare context. These actions would increase overall infection control efforts [6, 21, 36].

In this study, 46.2% of healthcare professionals recognized that NTM and MTB are clinically distinct. However, a larger proportion, 81.8%, believed that the GeneXpert test could detect both NTM and MTB. Conversely, many participants confused NTM with TB, with 67% suggesting that they are clinically similar. Additionally, 60% of respondents were unaware that acid‐fast bacillus (AFB) microscopy cannot differentiate between NTM and TB [1]. A recent global survey of physicians indicated that testing for NTM is typically triggered by certain clinical symptoms and underlying conditions, such as bronchiectasis. However, adherence to guidelines for NTM testing varies considerably across different regions, highlighting the necessity for more precise recommendations [37]. The lack of awareness and inconsistent testing practices can also lead to misdiagnosis and delayed treatment, contributing to increased morbidity associated with NTM infections. Enhanced training and systematic surveillance are recommended to improve HCWs' knowledge and response to NTM infections [33].

## 5 Strengths and Limitations of the Study

The prevalence and incidence of pulmonary NTM infections are increasing worldwide, particularly among vulnerable populations. Significant public health impacts have been observed, especially in resource‐limited settings, where inadequate healthcare systems hinder diagnosis and management. This lack of early intervention and prompt treatment for transmissible diseases exacerbates the issue. To our knowledge, this study is the first in Ethiopia to evaluate the knowledge of healthcare professionals regarding NTM infections. It has several strengths, including the identification of specific knowledge gaps among healthcare professionals in our region. Addressing these gaps can be achieved through educational enhancement initiatives, such as tailored training programs and ongoing professional development. In this under‐resourced setting, such public health interventions are crucial for improving the diagnosis and treatment outcomes of infectious diseases that are of high public health priority. Moreover, this study offers valuable insights into the current understanding of NTM infections among healthcare professionals in Ethiopia. It aims to assist local health policymakers in their strategic planning and resource mobilization for diagnosis and treatment, ultimately enhancing the quality of care. However, the study has its limitations; it is a single‐center, cross‐sectional study with a small sample size, which may not accurately reflect the national figure regarding knowledge gaps among healthcare workers in the country. This study reveals no significant associations between knowledge levels and demographic or clinical variables, opening avenues for future investigations. Furthermore, due to budget constraints, we are unable to provide additional training to address these critical knowledge gaps based on the key findings of this study.

## 6 Conclusions and Recommendations

This study highlights significant gaps in healthcare professionals' understanding of NTM infections, emphasizing the need for improved training. As NTM infections increasingly pose a public health concern, especially for immunocompromised individuals, HCWs should have a thorough knowledge of the related risk factors, clinical symptoms, diagnostic methods, and treatment options. The findings indicate that inadequate knowledge among healthcare professionals can result in misdiagnosis and suboptimal care, ultimately affecting patient outcomes. Addressing these knowledge gaps is particularly urgent given the rising prevalence of NTM infections among vulnerable populations.

To enhance the management of NTM infections, several targeted actions are recommended. It is crucial to implement focused training programs for healthcare professionals, especially in areas with high‐risk patient populations. These training sessions should cover disease epidemiology, clinical presentation, diagnostic techniques, and current treatment protocols. Additionally, establishing clear, evidence‐based protocols for the diagnosis and management of NTM infections will support accurate diagnosis and effective care. Regular updates to these protocols, incorporating the latest research and guidelines, will help ensure that healthcare providers remain informed. Furthermore, promoting collaboration among microbiologists, infectious disease specialists, and clinical practitioners can enhance both diagnosis and management. Organizing regular interdisciplinary workshops and case studies can facilitate knowledge sharing and improve diagnostic accuracy among HCWs.

## Author Contributions

Teklehaimanot Kiros conceived and designed the experiments, analyzed and interpreted the data, and wrote the paper. Mulat Erkihun, Bekele Sharew, Andargachew Almaw, Ayenew Assefa, Tegenaw Tiruneh, Birhanu Getie, Yenealem Solomon, and Shewaneh Damtie analyzed and interpreted the data, and wrote the paper.

## Ethics Statement

The authors have nothing to report.

## Conflicts of Interest

The authors declare no conflicts of interest.

## Data Availability

For this study, all data are presented within the manuscript without any restrictions.
